# Evaluating Adherence to Diabetic Retinopathy Care in an Urban Ophthalmology Clinic Utilizing the Compliance With Annual Diabetic Eye Exams Survey

**DOI:** 10.7759/cureus.34083

**Published:** 2023-01-23

**Authors:** Taylor S Davis, Faustine Luo, Sophie J Xie, Elena A Muro-Fuentes, Eduardo B Rodrigues

**Affiliations:** 1 Ophthalmology, Saint Louis University School of Medicine, St. Louis, USA; 2 Neurology, University of Iowa Hospitals and Clinics, Iowa City, USA

**Keywords:** annual eye examination, covid-19, panretinal photocoagulation, anti-vegf injections, adherence, diabetic retinopathy

## Abstract

Introduction

The objective of this study was to identify barriers that affect adherence to the management of diabetic retinopathy (DR) in an urban ophthalmology clinic. Patient beliefs regarding diabetic eye care, transportation to the eye clinic, the COVID-19 pandemic, and treatment with panretinal photocoagulation (PRP) or anti-vascular endothelial growth factor (anti-VEGF) injections were investigated.

Materials and methods

The original Compliance with Annual Diabetic Eye Exams Survey (CADEES) included 44 statements designed with a 5-point Likert scale to assess patients’ beliefs and understanding of their eye health and the importance of diabetic eye examinations. This survey was modified to include additional statements regarding the COVID-19 pandemic and free-response questions about transportation barriers and patients’ subjective experiences with PRP or anti-VEGF injections. A total of 365 patients with a diagnosis of any stage of DR from SLUCare Ophthalmology were identified as potential participants to complete the telephone survey. Patients were classified as non-adherent if they did not have a dilated eye examination within the past year, missed a scheduled follow-up appointment for DR care within the past year, or missed an appointment for anti-VEGF injections or PRP. The mean Likert scores for each CADEES statement were compared between the adherent and non-adherent groups using independent samples t-tests. Demographics and clinical indicators were also reported and compared between the two groups.

Results

Out of 365 patients, 68 completed the modified CADEES. Twenty-nine patients were adherent, and 39 patients were non-adherent. Results from six of the 54 CADEES statements were significantly different between the adherent and non-adherent groups. These statements addressed patients’ perception of their eye health, self-confidence in making an eye appointment, knowing someone with diabetic eye complications, self-confidence in controlling blood sugar, ability to use public transportation during the COVID-19 pandemic, and prioritizing eye health during the pandemic. There were no significant differences in clinical indicators or demographics between the adherent and non-adherent groups. Of the participants, 39.7% offered reasons for why transportation to the eye clinic was difficult. Patients suggested three novel reasons for missing eye appointments that were not specifically addressed in the CADEES. Fourteen unique barriers were reported for non-adherence with PRP or anti-VEGF injections.

Conclusions

The CADEES is a thorough tool for evaluating social barriers impacting adherence with DR appointments in an urban ophthalmology clinic. The survey did not identify any clinical or demographic risk factors for non-adherence in this patient population. Decreased patient self-efficacy may lead to non-adherence with the management of DR. The COVID-19 pandemic impacted the adherence of a small percentage of patients.

## Introduction

Diabetic retinopathy (DR) is a common complication of diabetes in which chronic hyperglycemia damages retinal blood vessels. The estimated prevalence of any form of DR is 34.6%, and the prevalence of vision-threatening DR is 10.2% [1]. For decades, panretinal photocoagulation (PRP) was the gold standard for the treatment of proliferative DR [2]. In recent years, however, several clinical trials demonstrated the effectiveness of anti-vascular endothelial growth factor (anti-VEGF) agents as superior alternative treatments for DR compared to PRP [2-4]. Injections with anti-VEGF antibodies have significantly reduced the cases of severe vision loss and other DR complications [3]. Despite the proven benefits of anti-VEGF treatment, loss to follow-up was reported as high as 20% in one clinical trial [3]. In clinical practice, adherence to any treatment is likely to be decreased compared to clinical trials, given that patient adherence is affected by an array of social factors. Therefore, identifying patients at risk for non-adherence with DR treatment may allow physicians to help these patients choose between PRP or anti-VEGF injections to optimize adherence.

The original Compliance with Annual Diabetic Eye Exams Survey (CADEES) was designed by Sheppler et al. to predict adherence to annual diabetic eye examinations [5]. Prior studies have investigated adherence to DR screening [6-10]. A study conducted in Shanghai, China, used the CADEES to predict adherence to annual diabetic eye examinations in patients with DR and identified many significant differences between the responses of adherent and non-adherent patients [11].

This study was designed to determine the social barriers, beliefs, and clinical indicators that may impact patient adherence with individual appointments for the management of DR in an urban ophthalmology clinic. Whereas prior studies have investigated adherence with annual diabetic eye examinations [5,11], we broadened our approach to include any missed appointments for DR care within a year. With this approach, we sought to understand the reasons patients miss scheduled follow-up appointments for the maintenance or treatment of DR. This study further aimed to assess the impact of the COVID-19 pandemic on adherence to eye appointments in patients with DR. Additionally, patients’ subjective experiences with both PRP and anti-VEGF injections were investigated.

## Materials and methods

This study was approved by the Saint Louis University Institutional Review Board (IRB) as an exemption (IRB #31090). A list of 365 patients with a diagnosis of any stage of DR was obtained from SLUCare Ophthalmology. Patients were included if they were 19-89 years of age, had a documented diagnosis of any stage of DR, and were last seen in the clinic after January 1, 2013.

We adopted the Compliance with Annual Diabetic Eye Exams Survey (CADEES), which was created by Sheppler et al. at the Devers Eye Institute [5]. The original CADEES included 44 statements that patients were asked to assess using a 5-point Likert scale (1 = strongly disagree, 2 = disagree, 3 = neither disagree nor agree, 4 = agree, 5 = strongly agree). The CADEES included patient demographics and clinical indicators. After reviewing the CADEES, items were added to assess specific barriers to follow-up for DR. Survey questions are denoted with a “Q” (e.g., Q3 = question 3), and CADEES statements are denoted with an “S” (e.g., S32 = CADEES statement 32). A screening question (Q2) was added to help categorize patients as adherent or non-adherent. Two questions (S20a and S20b) were added to identify the mode of transportation used and the difficulties of transportation. Statements regarding the COVID-19 pandemic (S45-S54) were included to determine its impact on adherence. Another series of questions was added to identify whether patients had received and adhered to treatment with anti-VEGF injections and/or PRP (Q7-Q7cii). A question about the type of diabetes was not included in the CADEES since the type of diabetes was obtained via chart review. The complete modified CADEES and telephone script used for this study can be found at https://github.com/med-pdf/uploads/raw/main/CADEES%20Phone%20Script.pdf.

Patients were recruited from June to October 2021 by calling during regular business hours (9 am to 5 pm) and inquiring if they would participate in a voluntary survey about the reasons patients do not attend follow-up visits for DR. Attempts to call patients were made a maximum of three times. Surveyors read a script that included a brief description of the survey length, content, and reasons for conducting the survey. Patients could complete the survey during the initial phone encounter or schedule a time to complete it later. The number and type of responses to telephone calls were recorded. All patients provided verbal consent before beginning the survey.

Patients were defined as non-adherent if they had not had a dilated eye examination within the past year (Q1), reported missing a scheduled follow-up appointment within the past year for DR (Q2), or reported missing an appointment for anti-VEGF injections or PRP (Q7aii and Q7cii). For the purposes of this study, missing a scheduled follow-up appointment within the past year for DR or missing an appointment for anti-VEGF injections or PRP was defined as not presenting to the clinic for a scheduled visit on any given day regardless if patients subsequently rescheduled visits for another date. The responses of adherent and non-adherent patients were compared using independent samples t-tests for all statements using a Likert scale (S1-S54). T-tests were used for the following clinical and demographic questions: Q3-Q6, Q8, Q10, and Q14. Chi-square analysis was performed for questions Q9, Q11-Q13, and type of diabetes. Differences between the adherent and non-adherent groups were deemed statistically significant if the two-sided p-value was less than 0.05 (p<0.05). Levene’s test for equality of variances was used for t-tests, and equal variances were assumed when the significance value was greater than 0.05 (significance>0.05). For questions with a Levene’s test less than or equal to 0.05, equal variances were not assumed, and the corresponding p-value was calculated and reported. Chi-square tests were performed using Pearson’s chi-square value, and results were considered significant if the asymptotic significance (two-sided) was less than 0.05. The Statistical Package for the Social Sciences (SPSS) for Macintosh version 28.0 (IBM SPSS Statistics, Armonk, NY, USA) was utilized to conduct all statistical tests.

A qualitative review of questions that elicited comments from patients was also conducted. S20a, S20b, S55, and Q7-Q7cii required recording of patient dialog. Surveyors typed the responses to these questions, and the responses were later categorized and analyzed.

## Results

Survey recruiting and adherence

**Figure 1:**
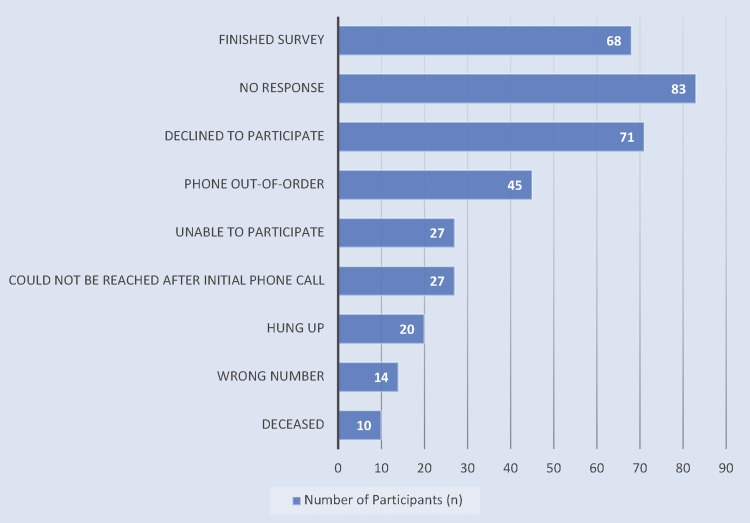
Phone Recruitment After three failed attempts to call a patient, the patient was marked as “No response.” Patients were considered “Unable to participate” if there was a language barrier, the participant denied being a patient of the clinic, the participant denied having DR during the initial recruitment phase, or the participant was otherwise not eligible to complete the survey. Participants who initially answered the phone but were lost to follow-up calls were categorized as “Could not be reached after the initial phone call.” Patients who immediately hung up after answering or during the explanation of the study were marked as “Hung up.” Calls that resulted in an individual answering but reporting a wrong number were placed in the “Wrong number” category. On occasion, those who answered the phone stated that the individual being contacted had passed away, and the patients were marked as “Deceased.” DR: diabetic retinopathy

Of 365 potential patients, 68 (18.63%) were recruited over the telephone and completed the modified CADEES, with 29 patients being adherent and 39 patients being non-adherent. Non-adherent patients could be deficient in one of the criteria for adherence or multiple criteria (e.g., missed a scheduled follow-up appointment and missed an appointment for anti-VEGF injections). Among the 39 non-adherent patients, 16 did not have a dilated eye examination within the past year, 25 missed a scheduled follow-up appointment, and 13 missed an appointment for anti-VEGF injections or PRP. The complete results of patient recruitment are found in Figure 1.

Adherent versus non-adherent CADEES statements

There were significant differences between the adherent and non-adherent groups for six of the 54 CADEES statements (S1, S4, S8, S30, S46, and S50). The mean Likert scores and p-values from t-tests comparing the adherent and non-adherent groups are included in Table 1 for all CADEES statements.

**Table 1:** CADEES Results *CADEES statements were considered statistically significant if the p-value was less than 0.05. †Equality of variances was assumed when Levene’s test for equality of variances was greater than 0.05. For statements with Levene’s test equal to or less than 0.05, the corresponding p-value was reported when the equality of variances could not be assumed. CADEES: Compliance with Annual Diabetic Eye Exams Survey, S: statement, n: number of participants

#	CADEES statement	Mean for adherent patients	Mean for non-adherent patients	p-value* (two-sided p)	Levene’s test for equality of variances - significance †	Adherent patients (n)	Non-adherent patients (n)
S1	My eyes are healthy.	2.93	1.97	0.007	0.098	29	39
S2	Early diabetic eye disease usually causes changes in vision.	3.97	4.21	0.351	0.374	29	39
S3	Having an eye examination is not pleasant.	2.97	2.97	0.977	0.667	29	39
S4	I am confident in my ability to make an appointment for an eye examination.	4.69	4.23	0.021	0.087	29	39
S5	Having an eye examination once a year can help me prevent losing my eyesight.	4.59	4.36	0.291	0.401	29	39
S6	I have trouble reading a book or newspaper, even if I use my glasses or contacts.	2.79	3.36	0.135	0.182	29	39
S7	Over the past four weeks, I have felt blue, downhearted, or depressed.	2.28	2.62	0.357	0.988	29	39
S8	I know someone who has lost some or all of his/her eyesight because of problems from diabetes.	2.72	3.62	0.032	0.050	29	39
S9	I know a lot about diabetes and the effect it can have on health.	4.41	4.44	0.907	0.300	29	39
S10	Diabetes can result in loss of visual function (e.g., difficulty reading and driving).	4.59	4.62	0.851	0.346	29	39
S11	I think I will lose some or all of my eyesight because of diabetes.	3.34	3.44	0.773	0.424	29	39
S12	I am confident I can keep a scheduled appointment with an eye doctor.	4.48	4.21	0.196	0.526	29	39
S13	I do not want to know if I have an eye disease.	1.52	1.62	0.691	0.942	29	39
S14	People who have good control of their diabetes are unlikely to have eye problems.	3.41	3.46	0.885	0.178	29	39
S15	Diabetes can cause severe eye problems.	4.55	4.38	0.375	0.217	29	39
S16	I would benefit from having an eye examination every year.	4.62	4.46	0.452	0.159	29	39
S17	My medical provider (i.e., doctor, nurse, and/or nurse practitioner) talks to me about the importance of eye examinations.	4.31	4.03	0.337	0.826	29	39
S18	Eye examinations cost too much.	2.72	3.00	0.425	0.911	29	39
S19	There is no treatment for diabetic eye diseases.	1.97	2.03	0.810	0.102	29	39
S20	It is hard for me to travel to an eye doctor.	1.86	2.41	0.106	0.151	29	39
S21	There are many things that make it hard to get an eye examination every year.	2.41	2.28	0.674	0.560	29	39
S22	I do not like having my eyes dilated with eye drops that make my pupils large.	2.90	3.26	0.281	0.946	29	39
S23	I think it is important to have an eye examination every year.	4.86	4.67	0.105	0.005	29	39
S24	My overall general health is excellent.	2.79	2.28	0.096	0.139	29	39
S25	Diabetic eye disease can be seen with an eye examination.	4.21	4.21	0.994	0.872	29	39
S26	Diabetes can damage the blood vessels in the eye.	4.62	4.72	0.434	0.091	29	39
S27	There are many eye doctors where I live.	3.52	3.41	0.735	0.790	29	39
S28	My family members or friends help me make doctor appointments.	2.66	3.18	0.180	0.866	29	39
S29	Eye examinations can find many different kinds of eye problems.	4.66	4.51	0.342	0.087	29	39
S30	I am confident I can control my blood sugar.	4.07	3.21	0.003	<0.001	29	39
S31	Having a yearly eye examination will help me save the eyesight I have now.	4.41	4.31	0.611	0.238	29	39
S32	People with diabetes are unlikely to get an eye disease.	1.90	1.85	0.846	0.157	29	39
S33	I cannot afford an eye examination.	2.03	2.13	0.749	0.301	29	39
S34	My insurance covers most of the cost of an eye examination.	4.14	4.05	0.760	0.580	29	39
S35	There are things I can do to prevent losing my vision from diabetes.	4.10	4.41	0.187	0.613	29	39
S36	Diabetic eye diseases often cause blindness.	4.14	4.33	0.333	0.970	29	39
S37	I have medical problems from diabetes.	4.03	4.38	0.165	0.793	29	39
S38	I want to get an eye examination every year.	4.55	4.41	0.465	0.133	29	39
S39	I only seek eye care when I am having trouble with my vision.	1.79	2.26	0.125	0.015	29	39
S40	Getting an eye examination every year is not one of my top priorities.	1.83	2.28	0.109	0.179	29	39
S41	I have an eye doctor I can go to for diabetic eye examinations.	0.97	0.87	0.150	0.005	29	39
S42	I receive a reminder from my eye doctor’s office when it is time to schedule an examination.	4.29	3.76	0.078	0.128	28	34
S43	I am happy with the care I get from my eye doctor.	4.46	4.29	0.468	0.907	28	34
S44	Visiting the eye doctor takes too much time.	2.54	2.65	0.735	0.669	28	34
S45	My family or friends were able to take me to my appointments during the COVID-19 pandemic.	4.05	4.07	0.939	0.719	21	27
S46	I was able to take public transportation to my appointment during the COVID-19 pandemic.	3.50	2.00	0.016	0.001	12	15
S47	I was able to travel to my appointment using a ride-share service during the COVID-19 pandemic.	3.33	2.88	0.438	0.297	12	17
S48	I was worried I would get COVID-19 if I went to the eye clinic.	1.93	2.03	0.732	0.425	29	39
S49	My eye clinic had available appointments during the pandemic.	4.10	3.74	0.165	0.486	29	39
S50	Maintaining my eye health has been a top priority during the pandemic.	4.24	3.56	0.017	<0.001	29	39
S51a	I missed an eye appointment because I was quarantining.	2.50	2.56	0.944	0.018	2	9
S52	I did not go to my eye appointment because I thought I may have had COVID-19.	1.55	1.87	0.221	0.449	29	39
S53	My treatment for diabetic retinopathy was important to me before the pandemic.	4.72	4.49	0.180	0.074	29	39
S54	My treatment for diabetic retinopathy has been important to me during the pandemic.	4.66	4.38	0.128	0.055	29	39

Clinical indicators and patient demographics

There were no significant differences between the adherent and non-adherent groups for any of the clinical indicators or demographics included on the CADEES. The results are shown in Table 2 and Table 3 based on the statistical test required for analysis. Given the small sample size, relationship status was divided by the presence or absence of social support into two groups for analysis: single/separated/divorced/widowed and married/domestic partnership. Similarly, patient education level was separated into two groups for analysis: high school graduate or less and some college or more. Our patient population was primarily composed of African American or Black and White patients, with only three patients identifying as another ethnicity (categorized as Other).

**Table 2:** Clinical Indicators and Patient Demographics (Independent Samples T-Test Analysis) Q: question, A1c: hemoglobin A1c, n: number of participants

#	Clinical indicator/demographic	Mean for adherent patients	Mean for non-adherent patients	p-value (two-sided p)	Levene’s test for equality of variances - significance	Adherent patients (n)	Non-adherent patients (n)
Q3	Years of diabetes	26.3846	25.4527	0.776	0.030	26	37
Q4	Know A1c	0.79	0.90	0.257	0.018	29	39
Q5	Last A1c level (%)	7.3722	7.9097	0.227	0.042	23	32
Q6	Years of diabetic retinopathy	12.100	8.353	0.155	0.059	20	34
Q8	Insurance status	1.00	0.97	0.393	0.082	29	39
Q10	Age	58.14	54.11	0.204	0.077	29	38
Q14	Total income ($)	70,469.50	41,834.31	0.132	0.014	24	26

**Table 3:** Patient Demographics (Chi-Square Analysis) Si: single, Sep: separated, Div: divorced, Wid: widowed, Marr: married, Dom P: domestic partnership, HSG: high school graduate, SC: some college, Afr Am: African American, n: number of participants

#	Demographic	Adherent patients (n)	Non-adherent patients (n)	Pearson chi-square	Asymptotic significance (two-sided) - p-value
Q9	Sex	Male = 13	Male = 16	0.098	0.754
		Female = 16	Female = 23		
Q11	Relationship status	Si/Sep/Div/Wid = 17	Si/Sep/Div/Wid = 24	0.143	0.706
		Marr/Dom P = 12	Marr/Dom P = 14		
Q12	Education	HSG or less = 6	HSG or less = 16	3.420	0.064
		SC or more = 23	SC or more = 22		
Q13	Race	Afr Am or Black = 14	Afr Am or Black = 21	0.806	0.668
		White = 11	White = 15		
		Other = 2	Other = 1		
—	Type of diabetes	Type 1 = 12	Type 1 = 13	0.463	0.496
		Type 2 = 17	Type 2 = 26		

Survey thoroughness

Participants were asked if they could provide additional reasons why patients do not have yearly eye examinations that were not covered in the CADEES (S55). The majority of patients’ responses had already been addressed by at least one statement. Three reasons suggested by participants were unique and not included in the original or modified CADEES. These reasons were scheduling conflicts due to work or family commitments, issues with the office management, and unexpected life events interfering with appointments.

Transportation (free-response questions)

S20a asked patients how they travel to the eye doctor. Twenty-five (36.76%) patients reported driving themselves, 41 (60.29%) reported having a friend or family member drive them, 11 (16.18%) reported taking private transportation (i.e., taxi or medical cab), and two (2.94%) reported taking a bus. Participants were allowed to report multiple ways they travel; therefore, the combined count and percentages do not equal the total number of participants (n = 68) nor 100%.

S20b asked patients to explain why it is difficult to travel to the eye clinic, and patients were allowed to offer more than one barrier. Forty-one (60.29%) patients stated that travel was not difficult. Eight (11.76%) patients cited lack of transportation or difficulty finding a ride as a barrier. Five (7.35%) patients had a physical disability that prevented them from driving. Four (5.88%) patients reported poor vision, and another four (5.88%) patients mentioned dilated eyes after their appointments. Four more (5.88%) patients cited other medical issues as a barrier to travel. Additional barriers to transportation included family responsibilities (n = 2, 2.94%), financial problems (n = 2, 2.94%), scheduling conflicts (n = 2, 2.94%), poor bus services (n = 1, 1.47%), parking at the hospital (n = 1, 1.47%), and difficulty traveling alone (n = 1, 1.47%).

Clinical responses about anti-VEGF injections and PRP

**Figure 2:**
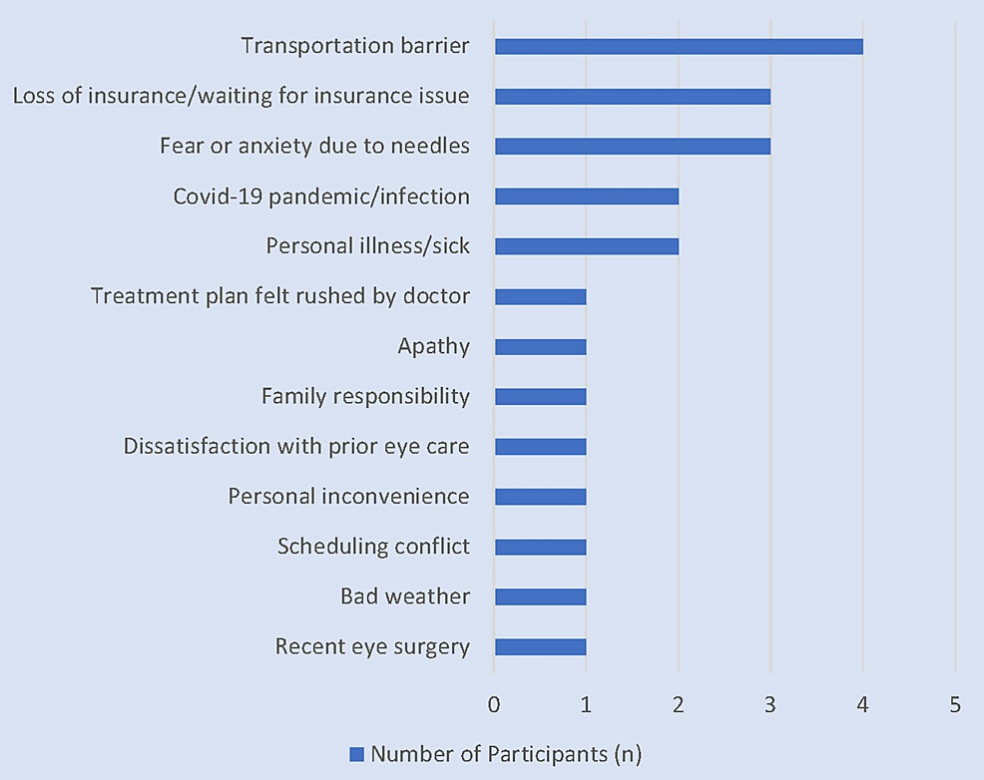
Barriers to Treatment With Anti-VEGF Injections or PRP Patients were allowed to give multiple reasons for missing appointments. VEGF: vascular endothelial growth factor, PRP: panretinal photocoagulation

Thirty-eight patients received anti-VEGF injections, 47 received PRP, and 18 denied having either treatment for DR. Eleven patients reported canceling or missing an appointment for their monthly anti-VEGF injections. The reasons for missing an appointment for injections included not having a ride (n = 3), anxiety/fear of needles (n = 3), insurance problem (n = 2), scheduling conflict (n = 2), COVID-19 infection (n = 1), knowing someone with an adverse event related to eye injections (n = 1), bad weather (n = 1), family member required assistance (n = 1), and avoiding eye injections due to recent eye surgery (n = 1). When specifically asked what prevented patients from following through with either treatment (Q7cii), participants suggested 14 unique explanations. Their responses are included in Figure 2.

Ten adherent and eight non-adherent patients never received any treatment for DR. Among the adherent patients, six of them reported that they were never told they needed treatment, and four of them reported that they were never diagnosed with DR. In the non-adherent group, six patients were never told they needed treatment, one patient reported never being diagnosed with DR, and one patient was recommended to have both injections and laser treatments but elected not to have either treatment.

## Discussion

Our modified CADEES identified six significant differences in the beliefs of adherent and non-adherent patients. The non-adherent group perceived their eyes as less healthy than the adherent group (S1), yet this did not encourage adherence to routine follow-up appointments. Our results differ from those of Zhu et al. who found that DR patients attending annual diabetic eye examinations perceived their eyes as less healthy than those who did not attend [11]. This disparity suggests that, in their population [11], perceiving one’s eyes as unhealthy could be a protective factor encouraging patients to attend routine eye examinations. However, this did not appear to be a protective factor for our population. Another study conducted in Taiwan found that patients who believed that their eyes were healthy were less likely to adhere to annual diabetic eye screening [6].

The non-adherent group was less confident in their ability to make an eye appointment (S4). This suggests that patient self-confidence plays a role in adherence to eye care. Similarly, adherent patients reported greater confidence in their ability to control their blood sugar (S30). Perceived self-efficacy in managing blood sugar levels may suggest an increased likelihood that patients will follow up for the management of DR.

The non-adherent group was more likely to have known someone who lost vision due to diabetes (S8). This is surprising and unexpected, since one may hypothesize that having anecdotal evidence of severe visual complications related to diabetes would encourage individuals with DR to monitor their vision more closely.

We added 10 statements to our modified CADEES to assess the impact of the COVID-19 pandemic on adherence to appointments. We found a statistically significant difference between the adherent and non-adherent groups regarding the ability to use public transportation during the pandemic (S46). However, only two participants reported using the bus for transportation (S20a). This may be explained by patients choosing to select one of the disagree options for S46 if they never used public transportation rather than choosing the “Not applicable” option. We observed that 13% of the patients worried that they would be infected with COVID-19 if they went to the eye clinic (agreed or strongly agreed on S48). This finding is similar to another study in which 14% of the participants contacted via telephone believed that it was likely or extremely likely that they would be exposed to COVID-19 at eye visits [12]. In addition, two patients cited the COVID-19 pandemic as the cause for not following up for treatment with anti-VEGF injections or PRP (Q7cii). Overall, the pandemic prevented a small percentage of our surveyed patients from complete adherence to DR care.

Non-adherent patients were significantly less likely to agree that maintaining their eye health was a top priority during the pandemic (S50); however, the non-adherent group overall agreed that their treatment for DR was important before and during the pandemic (S53 and S54). This suggests that these patients may have been less adherent to DR care during the pandemic due to other stressors or altered priorities.

Social determinants of health such as income, social support, and transportation are known to affect lifespan and overall health outcomes [13]. In one study assessing loss to follow-up in patients receiving anti-VEGF injections or PRP for proliferative diabetic retinopathy, younger age, non-White race, and lower regional average adjusted gross incomes were identified as risk factors for loss to follow-up [14]. In comparison, our study revealed no significant differences between adherent and non-adherent patients regarding income, health insurance status, education level, relationship status, sex, age, or race. Our findings may differ from those of Obeid et al. due to our smaller population size [14]. Although not statistically significant, there appears to be an income gap between adherent (mean income = $70,470) and non-adherent (mean income = $41,834) patients in our study. One limitation of this study is that the CADEES did not consider baseline wealth. Some affluent, retired individuals may have reported no annual income or smaller annual incomes, which masks a patient’s underlying wealth. Subsequent studies using the CADEES should seek to determine annual salary prior to retirement.

No clinical indicators (i.e., type of diabetes, years of diabetes, years of DR, and last A1c) were significantly different between adherent and non-adherent patients. In contrast, Zhu et al. found that DR patients were more likely to be non-adherent with annual diabetic eye examinations if they had a shorter duration of diabetes, better visual acuity on presentation, less severe DR, less education, and poor glucose control [11]. Our lack of clinical indicators predictive of adherence may be due to our relatively small sample size. However, our study aligns with prior studies that showed no correlation between adherence and annual diabetic eye examinations and type of diabetes, age, or sex [5,11].

Transportation is recognized as a substantial barrier leading to non-adherence with follow-up appointments and medical treatment [15]. We also discovered that transportation was a prominent barrier to maintaining appointments among DR patients. Of the total participants, 22% reported difficulty traveling to the eye clinic (S20); however, when asked why traveling to the eye clinic was difficult (S20b), 39.7% of the participants provided explanations regarding the difficulties of transportation. Our findings are consistent with previous research evaluating transportation as a barrier to healthcare access [15].

We asked patients to recollect their experiences receiving anti-VEGF injections and/or PRP, including any reasons for not attending appointments. The most commonly reported barriers by non-adherent patients were lack of a ride (n = 3) and anxiety/fear of injections (n = 3). Müller et al. developed a novel questionnaire to evaluate barriers to intravitreal therapy and similarly found that 14.5% of patients were impacted by fear of the treatment or its side effects [16]. Fear of injections is a significant barrier that physicians must consider when proposing this therapy to patients. Two patients missed appointments for injections due to insurance issues. Notably, only one participant denied having insurance. According to the 2020 United States Census, 8.6% of the US population did not have health insurance coverage [17]. Zhang et al. similarly observed in a large population study that 8.2% of Americans with visual problems did not have insurance [18]. Thus, our results may not fully represent the barrier that lack of insurance imposes on patients with DR.

Our modified CADEES is a thorough tool for identifying risk factors for non-adherence with appointments in patients with DR. When asked if there were other reasons why patients may not attend yearly eye examinations (S55), 41 participants attempted to provide explanations not covered in the CADEES, but only three explanations were not already addressed in the survey. We suggest that future studies using the CADEES include statements to assess how scheduling conflicts due to work or family commitments and unexpected live events interfere with adherence to DR care. Additionally, the modified CADEES included free-response questions that encouraged dialog to better understand the difficulties faced by patients. Participants were willing to discuss factors that made it difficult for them to attend appointments. Future patients may benefit from physicians exploring these barriers with them.

Our study was not without limitations. Only 18.63% of the potential survey participants completed the survey. The CADEES required 25 minutes to complete, and many patients likely declined to participate given the length of the survey. It is probable that recruitment and the completion of the CADEES in the clinic would have resulted in a higher completion rate. However, patient outreach over the telephone enabled us to recruit non-adherent patients who had not had an eye examination within the past year and would have likely not presented to the clinic during our recruitment period. Another limitation is the survey’s self-reported nature. We asked patients to recall information that may not be easily remembered, such as the last hemoglobin A1c and years since being diagnosed with DR. The results are subject to memory and patients being willing to admit to missing appointments. Furthermore, our study did not attempt to determine the duration for which patients were non-adherent. Our objective was to identify potential barriers that prevent patients from attending any appointment at the eye clinic for DR care. Additional studies may attempt to determine the duration of non-adherence among patients with DR and how the barriers observed in this study correlate to the length of non-adherence.

## Conclusions

The modified CADEES provides a thorough assessment of the barriers to attending appointments for the management of diabetic retinopathy (DR) in an urban ophthalmology clinic. Our modified CADEES identified several significant differences between the beliefs of adherent and non-adherent patients, while also ascertaining specifics about transportation barriers, the impact of COVID-19 on adherence with DR eye appointments, and difficulties with anti-VEGF injections and PRP. Demographics and clinical indicators did not differ significantly between adherent and non-adherent patients. Decreased patient self-efficacy may promote non-adherence with DR management. Transportation was a common barrier for patients. The COVID-19 pandemic prevented a small percentage of surveyed patients from being adherent. Clinics should attempt to identify and address risk factors for non-adherence with DR appointments, such as patient self-efficacy and transportation.
